# Association between Biofilm-Production and Antibiotic Resistance in Uropathogenic *Escherichia coli* (UPEC): An In Vitro Study

**DOI:** 10.3390/diseases8020017

**Published:** 2020-06-07

**Authors:** Payam Behzadi, Edit Urbán, Márió Gajdács

**Affiliations:** 1 Department of Microbiology, College of Basic Sciences, Shahr-e-Qods Branch, Islamic Azad University, Tehran 37541-374, Iran; behzadipayam@yahoo.com; 2 Department of Public Health, Faculty of Medicine, University of Szeged, 6720 Szeged, Dóm tér 10, Hungary; tidenabru@freemail.hu; 3 Institute of Translational Medicine, University of Pécs Medical School, 7624 Pécs, Szigeti utca 12, Hungary; 4 Department of Pharmacodynamics and Biopharmacy, Faculty of Pharmacy, University of Szeged, 6720 Szeged, Eötvös utca 6, Hungary; 5 Institute of Medical Microbiology, Faculty of Medicine, Semmelweis University, 1089 Budapest, Nagyvárad tér 4, Hungary

**Keywords:** urinary tract infections, uropathogen, *Escherichia coli*, biofilm, colony morphology, antibiotic, resistance, crystal violet

## Abstract

Urinary tract infections (UTIs) are among the most common infections requiring medical attention worldwide. The production of biofilms is an important step in UTIs, not only from a mechanistic point of view, but this may also confer additional resistance, distinct from other aspects of multidrug resistance (MDR). A total of two hundred and fifty (*n* = 250) *Escherichia coli* isolates, originating from clean-catch urine samples, were included in this study. The isolates were classified into five groups: wild-type, ciprofloxacin-resistant, fosfomycin-resistant, trimethoprim-sulfamethoxazole-resistant and extended spectrum β-lactamase (ESBL)-producing strains. The bacterial specimens were cultured using eosine methylene blue agar and the colony morphology of isolates were recorded. Antimicrobial susceptibility testing was performed using the Kirby–Bauer disk diffusion method and E-tests. Biofilm-formation of the isolates was carried out with the crystal violet tube-adherence method. *n* = 76 isolates (30.4%) produced large colonies (>3 mm), mucoid variant colonies were produced in *n* = 135 cases (54.0%), and *n* = 119 (47.6%) were positive for biofilm formation. The agreement (i.e., predictive value) of mucoid variant colonies in regard to biofilm production in the tube-adherence assay was 0.881 overall. Significant variation was seen in the case of the group of ESBL-producers in the ratio of biofilm-producing isolates. The relationship between biofilm-production and other resistance determinants has been extensively studied. However, no definite conclusion can be reached from the currently available data.

## 1. Introduction

The global burden of diseases has shown considerable changes in the last century. Despite global trends due to demographic and epidemiological transitions, and the more pronounced role of non-communicable illnesses, infectious pathologies are still an important factor for morbidity and mortality [1]. Among these infectious diseases, urinary tract infections (UTIs) are some of the most common illnesses caused by pathogenic microorganisms, predominantly by facultative Gram-negative bacteria [2,3,4]; the most common causative agents of UTIs are the members of the *Enterobacterales* order [5,6]. More specifically, the principal cause of UTIs (>90%) are uropathogenic *Escherichia coli* (UPEC) and uropathogenic *Klebsiella pneumoniae* (UPKP), while other members of the order are represented to a lesser extent [7,8]. These infections account for 10–30% of infections in community settings and 25–60% of nosocomial infections overall (predominantly due to catheter-associated infections), representing a serious economic and public health issue for healthcare infrastructures [9,10]. Depending on the extent of the infection, UTIs may affect the tissues of the lower urinary tract, urethra, bladder and the kidneys, and be classified either as uncomplicated or complicated infections; in severe cases, UTIs may be the sources of bacteremia and urosepsis [11]. UTIs are associated with important societal and monetary considerations: they are associated with approximately 10 million general practitioner (GP) visits, 1.5 million emergency room visits and 300,000 hospital admissions in the United States alone [12]. The subsequent costs of UTIs for national economies (including losses due to sick leave, hospital admissions and pharmacotherapy) may be pronounced, estimated to be as high as USD 6 billion [13]. 

The therapy of UTIs largely depends on the anatomical regions affected (acute uncomplicated cystitis vs. pyelonephritis), the anamnestic data of the patients (e.g., age, gender, underlying conditions, pregnancy, hypersensitivity) and the results of culture/susceptibility data, if available [14,15]. Based on current international guidelines, nitrofurantoin, fosfomycin, trimethoprim/sulfamethoxazole and mecillinam are recommended first-line agents for the therapy of uncomplicated UTIs [14,15,16]. In case of complicated infections or patient characteristics for complicated UTIs, drug allergies or extensive resistance to the abovementioned drugs, third generation cephalosporins, carbapenems and aminoglycosides may also be considered [17]. Fluoroquinolones have previously been used as first-line agents in the therapy of UTIs. However, due high levels of resistance in Gram-negative bacteria, the concept of “collateral damage” (affecting the gastro-intestinal and vaginal flora) and the subsequent risk of *Clostridioides difficile* infections (CDIs) and recent developments in the adverse events associated with these drugs (including the “black box” warning labels added by the Food and Drug Administration), the importance of these agents has decreased significantly, only to be used in well-defined indications [18,19]. 

One of the most important issue associated with the therapy of UTIs is the emergence and spread of multidrug-resistant (MDR) bacterial strains-owing to a plethora of different resistance mechanisms-severely limiting therapeutic alternatives for clinicians [20,21,22]. This is especially daunting, if urinary pathogens also possess intrinsic resistance mechanisms or patients have some underlying conditions [8,23]. High levels of resistance against urinary antibiotics have been reported in Gram-negative bacteria in regions where no restrictions were introduced to their use in the community [24]. In addition to these resistance mechanisms, the production of biofilms is another major concern, often leading to therapeutic failure [25]. Biofilms are aggregates of bacterial communities inside an extracellular matrix, composed from exopolysaccharides (EPS), proteins and nucleic acid produced by the multiple bacterial species [26]. Biofilms allow for bacterial communities to attach to various inanimate and in vivo environments, and provide protection for these bacteria from harsh environmental conditions and noxious agents, such as antibiotics [25,26]. In fact, the minimal inhibitory concentration of antibiotics against bacteria embedded in biofilms may be 10^1^–10^4^-times higher, than against planktonic cells [27]. The eradication of biofilm-embedded pathogens in vivo is still an important concern, as there are no specific drugs available for the eradication of these bacteria in infections [28]. The composition of biofilms is inherently heterogenous (including physiological heterogeneity, genetic variability and stochastic events in gene expression), which makes it difficult to study them in experimental conditions. Nevertheless, there are various methods available for their characterization, including staining methods, chromogenic and experimental media, plate-based methods evaluated by spectrophotometric measurements, electron microscopy and flow chamber systems [25,26,27,28]. These methods vary considerably in their reproducibility, price and the adaptability of the resulting data to in vivo conditions; nevertheless, due to the significance of biofilm-production in clinical syndromes and antibiotic non-susceptibility, all of them are frequently used [25,26,27,28,29,30]. 

The role of biofilm-formation during the pathogenesis of UTIs (especially in catheter-associated infections) has been extensively studied: production of this extracellular matrix aids in the attachment, provides protection against the sheer forces in the urinary tract and promotes bacterial persistence and chronicity [29,30]. 

Recently, several studies aimed to assess the relationship between the phenotypic characteristics of bacterial pathogens, biofilm-formation and antibiotic-resistance [31]. However, these studies have often provided conflicting results. The aim of our present study was to assess the potential correlation between the resistance characteristics of UPEC species and their biofilm-forming capacity in laboratory-based study.

## 2. Materials and Methods

### 2.1. Collection of Isolates

A total of two hundred and fifty (*n* = 250) *E. coli* isolates, which were kindly provided by various Hungarian hospitals, were included in this study. The isolates originated from clean-catch urine samples from patients with laboratory-confirmed UTIs. The *n* = 250 isolates included wild-type/pan-susceptible strains (control group), strains resistant to ciprofloxacin (CIP group), strains resistant to fosfomycin (FOS group), strains resistant to trimethoprim-sulfamethoxazole (SXT group) and extended spectrum β-lactamase (ESBL)-producers in equal measure (*n* = 50 strains in each group). The isolates in the latter four groups were selected specifically on the basis that they are not resistant to other agents in any of the other tested antibiotic groups. During our experiments, *E. coli* ATCC 25298 (pan-susceptible, “wild strain”, strong biofilm-producer [32]), *E. coli* ATCC 35218 (*bla*_TEM-1_-producer, weak biofilm-producer [33]; obtained from the American Type Culture Collection, Manassas, VI, USA), *E. coli* 15/12569 (resistant to ciprofloxacin; MIC_ciprofloxacin_ = 2 mg/L), *E. coli* 17/47012 (resistant to fosfomycin; MIC_fosfomycin_ = 64 mg/L) and *E. coli* 16/30098 (resistant to trimethoprim-sulfamethoxazole; MIC_SXT_ = 16 mg/L) were used as control strains (clinical isolates obtained from various hospitals).

### 2.2. Bacterial Identification

Identification of *E. coli* isolates was carried out using matrix-assisted laser desorption/ionization time-of-flight mass spectrometry (MALDI-TOF MS; Bruker Daltonics, Bremen, Germany). Bacterial cells from fresh overnight cultures were transferred to a stainless-steel target. An on-target extraction was performed by adding 1 µL of 70% formic acid prior to the matrix. After drying at an ambient temperature, the cells were covered with 1 µL matrix (α-cyano-4-hydroxy cinnamic acid in 50% acetonitrile/2.5% trifluoro-acetic acid; Bruker Daltonics, Bremen, Germany). MALDI-TOF MS measurements were performed by the Microflex MALDI Biotyper (Bruker Daltonics, Bremen, Germany) in positive linear mode across the *m*/*z* range of 2 to 20 kDa; for each spectrum, 240 laser shots at 60 Hz in groups of 40 shots per sampling area were collected [34]. The MALDI Biotyper RTC 3.1 software (Bruker Daltonics, Bremen, Germany) and the MALDI Biotyper Library 3.1 were used for spectrum analysis. Based on spectrum analysis, a log(score) value was provided, indicating the reliability of MALDI-TOF MS identification: a log(score) <1.69 showed unreliable identification, 1.70–1.99 corresponded to probable genus-level identification, 2.00–2.29 corresponded to reliable genus-level identification, while a score ≥2.30 corresponded to reliable species-level identification [31]. All isolates included in the study were re-identified as *E. coli* before further experiments.

### 2.3. Colony Characteristics

The bacterial specimens were cultured using eosine methylene blue (EMB) agar (bioMérieux, Marcy-l’Étoile, France) plates. To record colony morphology of the bacterial isolates, EMB plates were inoculated and incubated at 37 °C for 24 h, at aerobic atmosphere. After the incubation period, colony morphologies were assessed visually (for size, mucoid nature and lactose-fermentation) and these data were recorded. Colonies were considered small if their side was below 3 mm, or large if their size was >3 mm [33].

### 2.4. Antimicrobial Susceptibility Testing, Resistance Detection

Antimicrobial susceptibility testing (AST) was performed either using the Kirby–Bauer disk diffusion method and E-tests (Liofilchem, Abruzzo, Italy) on Mueller–Hinton agar (MHA) plates. The interpretation of the results was based on EUCAST breakpoints v. 9.0. (http://www.eucast.org). *E. coli* ATCC 25298 was used as a quality control strain. Screening for ESBL-production was carried out using cefpodoxime 10 µg disks; the screening test was considered positive, if the inhibition zone diameter was <21 mm [35]. Verification of ESBL-production was performed using the AmpC-ESBL Detection Set (MAST Diagnostica GmbH, Reinfeld, Germany) [7]. 

### 2.5. Assessment of Biofilm-Production by the Tube-Adherence Method

Biofilm-formation of the isolates was carried out in the tube-adherence method described previously [36]. Briefly, glass tubes containing 1 mL of sterile trypticase soy broth (bioMérieux, Marcy-l’Étoile, France) were inoculated with 1 µL of the overnight culture of a respective bacterial strain. The tubes were then incubated statically for 24 h at 37 °C. Verification of planktonic growth was observed visually. After the incubation period, the supernatant was then discarded, the adhered cells were rinsed three times with phosphate buffer saline (PBS; Sigma-Aldrich; Budapest, Hungary) and the tubes were patted dry on a paper towel. The contents of the tubes were treated with a 1 mL solution of 0.1% crystal violet (CV; Sigma-Aldrich; Budapest, Hungary) to stain the adhered biomass; the tubes were incubated for 3 h at room temperature with the staining solution. The crystal violet solution was then discarded and the tubes were again rinsed three times with PBS and the tubes were patted dry on a paper towel. Biofilm-formation was observed visually; the appearance of visible biofilm lining at the bottom and on wall of the glass tubes were considered positive for biofilm-production [36].

### 2.6. Statistical Analysis

Descriptive statistical analysis (including means and percentages to characterize data) was performed using Microsoft Excel 2013 (Microsoft Corp.; Redmond, WA, USA). Additional statistical analyses were performed with IBM SPSS Statistics for Windows 24.0 (IBM Corp., Armonk, NY, USA), using the χ^2^-test. *p* values < 0.05 were considered statistically significant. Additionally, consistency-assessment was also performed between the results of the colony morphology and biofilm-production studies [31]. 

### 2.7. Ethical Considerations

Clinical, personal and epidemiological data pertaining to the affected patients was not collected or provided during the study, bacterial isolates were only identifiable based on their serial number; therefore, our present study was not subject to ethics review. 

## 3. Results

### 3.1. Association of Antibiotic Resistance with Colony Characteristics in Uropathogenic E. coli (UPEC)

**Figure 1:**
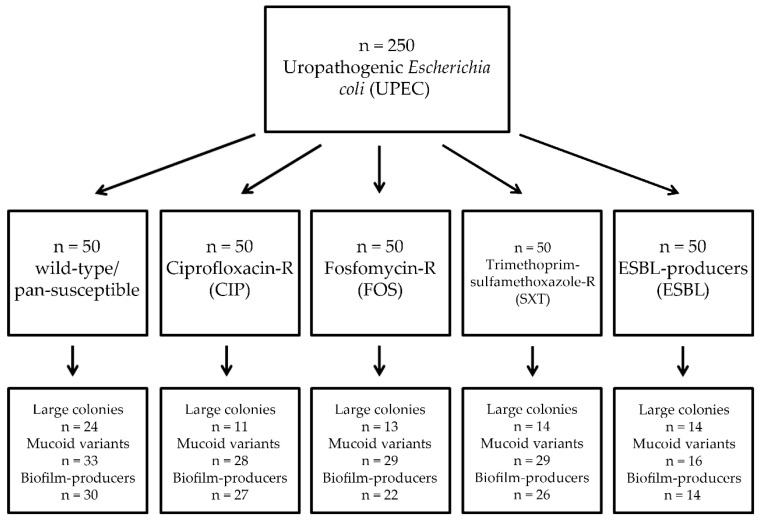
Summary of the experimental results in the different groups of uropathogenic *Escherichia coli* (UPEC)**.**

Out of the *n* = 250 *E. coli* isolates included in this study, *n* = 76 isolates (30.4%) produced large colonies (>3 mm): *n* = 24 in the control group, while this was *n* = 11 (*p* = 0.0064; χ^2^ = 7.428; degrees of freedom [DOF]: 1) in the CIP-group, *n* = 13 (*p* = 0.023; χ^2^ = 5.191; DOF: 1) in the FOS-group and *n* = 14 (*p* = 0.039; χ^2^ = 4.245; DOF: 1) in both the SXT- and ESBL-producer group. The emergence of large-colony producing isolates was significantly lower in all antibiotic-resistant groups, compared to the wild type isolates (Figure 1).

Mucoid variant colonies were produced in *n* = 135 cases (54.0%): in the control group, 33/50 isolates showed mucoid colonies, while this ratio was 28/50, 29/50 and 29/50 for the CIP-group, SXT-group and the FOS-group, respectively (*p* > 0.05 in all cases) (Figure 1). Significant variation was seen only in the case of the group of ESBL-producers (16/50 isolates; *p* < 0.0001; χ^2^ = 11.561; DOF: 1). Most *E. coli* isolates (98.8%) showed lactose-fermentation when observed on EMB-agar after 24 h, only *n* = 3 (one in the ESBL-group and two in the CIP-group) lactose-non fermenting isolates were noted. Among the control strains, *E. coli* ATCC 25298 presented with small, mucoid colonies, *E. coli* ATCC 35218, *E. coli* 15/12569, *E. coli* 17/47012 with small, non-mucoid colonies, while *E. coli* 16/30098 with large, non-mucoid colonies. All control strains were lactose-fermenters.

### 3.2. Association of Antibiotic Resistance with Biofilm-Formation in UPEC

Out of the tested isolates, *n* = 119 (47.6%) were positive for biofilm formation in the tube-adherence assay: in the control group, 30/50 isolates showed biofilm-formation, while this ratio was 27/50, 26/50 and 22/50 for the CIP-group, SXT-group and the FOS-group, respectively (*p* > 0.05 in all cases). Pronounced differences were observed for the ESBL-group, where only 14/50 isolates produced biofilm in this assay (*p* = 0.0013; χ^2^ = 10.39; DOF: 1) (Figure 1). The agreement (i.e., predictive value) of mucoid variant colonies in regard to biofilm production in the tube-adherence assay was 0.881 or 88.1% overall (control: 0.909, CIP: 0.964, SXT: 0.897, FOS: 0.759, ESBL: 0.875). Among the control strains, *E. coli* ATCC 25298 was positive for biofilm-formation, while all other control strains were negative. 

## 4. Discussion

UTIs caused by drug-resistant *E. coli* is an important clinical concern, affecting a large amount of patients, both in outpatient and in hospital settings [37]. Recent literature reports highlight that the resistance levels of uropathogenic *E. coli* against fluoroquinolones, trimethoprim-sulfamethoxazole and third generation cepalosporins in Iran is ranging between 30–60%, 40–80% and 15–66% [38]; while this ratio in Hungary is between 20–40%, 5–15%, 25–40%, 10–30%, and 10–15% for fluoroquinolone, nitrofurantoin, trimethoprim-sulfamethoxazole, third generation cephalosporin, and fosfomycin-resistant isolates, respectively [21]. Catheter-associated UTIs (CA-UTIs) are the most nosocomial infections worldwide, leading to high costs for the healthcare-providers and decreased quality of life for the affected patients [9,39]. The production of biofilms is ubiquitous in the environment and has been described in 65% of human infections (affecting patients from all backgrounds and in all age groups); therefore, they present an important unresolved issue for both clinicians and researchers [40]. The characteristic of producing EPS matrix also strongly correlates to the proclivity of these bacteria to become nosocomial pathogens (*K. pneumoniae*, *Pseudomonas aeruginosa*, *Acinetobacter* spp., *Staphylococcus aureus* among others), surviving in the harsh physical environments of hospitals [41,42,43,44]. Biofilms also contribute to bacterial antibiotic resistance in an indirect manner, by augmenting the pharmacokinetic properties (i.e., the ability of these drugs to penetrate and reach the microorganisms in effective concentrations) of the anatomical region [25,26,27,28,29,30]. The production of biofilms also correspond to transcriptional changes in these bacteria (often mediated by quorum-sensing [QS]-based mechanisms); this may lead to the differential expression of various virulence factors, metabolic end-products or antibiotic-resistance determinants [45]. Bacterial cell–cell communication (or QS) is the phenomenon of transcriptional changes in bacteria due to reaching a threshold in surrounding bacterial population density, mediated by secreted compounds termed autoinducers [45]. As biofilm-formation is a mutually-beneficial, cooperative behavior to enhance the survival of the overall bacterial population, it is not surprising that genes involved in biofilm-development are QS-mediated, highlighting the close interdependence of the two mechanisms [45,46]. Comparisons between biofilm and planktonic cells are difficult to make due to the intrinsic difference between the two modes of growth, such as compositional differences in biomass. These evolutionary trade-offs may explain differential resistance-trends in the same species of biofilm-producing and non-producing bacteria during in vitro testing [46]. 

In our present study, *n* = 250 *E. coli* isolates were characterized, using phenotypic tests to ascertain a possible correlation between resistance and biofilm-formation. Our isolates were grouped based on their distinct resistance mechanisms and compared to the control group, containing wild-type *E. coli*. 62.0% of isolates overall produced mucoid colonies, and almost half (47.6%) of the tested isolates produced biofilm in the tube-based assay. Based on our results, the predictive power of mucoid-variant colonies for positivity in the biofilm-assay was 88.1%; our results contrast the findings of Whelan et al., who noted 50 out of 62 (81%) tested *E. coli* isolates as strong biofilm-producers and a much lower (4%) predictive power between mucoid-type *E. coli* on Cysteine Lactose Electrolyte Deficient (CLED) agar and strong biofilm-formation [31]. Lajhar et al. have come to similar conclusions, when studying the biofilm-formation of *E. coli* O26 pathotypes in different in vitro settings and on various surfaces [47]. Significant differences in the share of mucoid colony-forming and biofilm-producing strains *E. coli* were only observed in the group of ESBL-producers (positivity was much lower, compared to the control group), while such differences were not shown for the CIP, FOS and SXT-groups. On the other hand, differences in colony size were noted between the control group all other isolates, showing any kind of phenotypic resistance. The ratio of lactose-non-fermenting *E. coli* in our study was 1.2% (*n* = 3), which is similar to the global prevalence of lactose-negative strains (0–5%) reported in other studies [48]. The number of lactose-negative isolates in the present study was too low to provide any relevant conclusions on the topic. Nevertheless, in another study by Gajdács et al., the antibiotic susceptibilities of lactose-fermenting and non-fermenting *E. coli* from UTIs were compared during a 5-year study period in Southern Hungary. In this study, it was found that the resistance rates of lactose non-fermenters were higher for fluoroquinolones, fosfomycin and nitrofurantoin in both inpatients and outpatients [49].

Although the correlation between biofilm-production and the expression of non-biofilm-based antibiotic resistance has been a topic of pronounced interest in the last several years, the literature surrounding this topic contains conflicting data, therefore definite conclusions cannot be drawn as of now. In a study by Dumaru et al., the correlation between the MDR phenotype, ESBL- and carbapenemase-production and biofilm-formation were assessed in gut bacteria and non-fermenters [36]; in their report, 62.7% of isolates were biofilm-producers and there was strong association found between the MDR-status, carbapenemase-production and biofilm-production, while this was not shown for ESBL-positivity. Nirwati et al. studied the potential association of resistance and biofilm-production in *n* = 167 *K. pneumoniae* isolates: 55.7% of their tested isolates were biofilm-producers (moderate or strong), and in the non-biofilm-producing group, resistant isolates (both non-MDR and MDR) were less common, then among biofilm-positive isolates [50]. Avila-Novoa et al. studied the correlation between biofilm-formation and MDR in *A. baumannii* using phenotypic and genotypic methods; in their study, 73.3% of isolates were biofilm-producers based on the Congo red agar method and showed a 73.3% susceptibility to cefepime and a 53.3% susceptibility to ciprofloxacin. Nevertheless, their study did not find any clear association between biofilm-production and susceptibility [51].

The study of Cepas et al. included various Gram-negative bacteria (*E. coli*, *K. pneumoniae*, and *P. aeruginosa*), which found no direct link between possessing the MDR phenotype and biofilm-production. However, individual associations with resistance to several antibiotics (gentamicin and ceftazidime in *E. coli*, ciprofloxacin in *P. aeruginosa* and colistin in *K. pneumoniae*) and biofilm-positivity were noted [52]. In the study performed by Qi et al. the phenotypic and genotypic characterization and typing of *A. baumannii* isolates were performed, in the context of biofilm-production [53]; in their analysis of *n* = 272 isolates, they revealed that non-MDR isolates were more common in biofilm-non-producers. The correlation of meropenem-resistance and the ability to form biofilms were assessed by Perez et al. [54], where an inverse relationship was identified between carbapenem non-susceptibility and biofilm-production. These results were further verified by Mustafer et al., where *P. aeruginosa* strains showing imipenem-resistance (owing to a variety of resistance mechanisms) [55]. In addition, Fábréga et al. also showed an inverse relationship between biofilm-production and quinolone-resistance, in the context of *Salmonella enterica* [56]. In contrast, Gurung et al. showed that MDR *P. aeruginosa* and *A. baumanni* strains were significantly more common among strong biofilm-producers, compared to the non-biofilm group [57]. Regarding UPEC strains, the article published by Neupane et al. showed that biofilm-production in ESBL-producing *E. coli* correlated with more extensive biofilm-production, compared to susceptible isolates [58], while Soto et al. noted an inverse correlation between biofilm-production and quinolone resistance [59]. In a longitudinal cohort study, Bartoletti et al. found that in their “Chronic Bacterial Prostatitis (CBP) population”, biofilm-producing bacteria were commonly found and had a significant negative impact on the clinical response to the adequate antibiotic therapy [60]. Thus, it may be concluded that there may be some kind of association between the expression of antibiotic-resistance-determinants and biofilm-production. However, the exact nature of that relationship still needs further studies [61,62].

## 5. Conclusions

UTIs are one of the most common infections requiring medical attention worldwide. The therapy of UTIs caused by *E. coli* may be hindered by the extensive resistance levels associated with these agents. The production of biofilms is an important step in UTIs, not only from a mechanistic/pathogenetic point of view, but this may also confer additional resistance through a mechanism distinct from other aspects of MDR. In fact, the relationship between biofilm-production and other resistance determinants have been extensively studied. However, no definite conclusion can be reached by the currently available data. Our study aims to provide additional data to the present knowledge base on the topic. While the phenotypic characteristics of UPEC colonies were shown to be affected by the presence of any resistance mechanisms (compared to wild-type strains), this statistical association was not shown in the case of biofilm-production, except for the case of ESBL-producers, where biofilm-producers were significantly less common. While other studies noted associations between the MDR phenotype or resistance against various antibiotics and biofilm-production, only a numerical, but not statistical difference was shown in our study. In contrast, while other studies showed poor correlation between colony characteristics and biofilm-production, a >85% agreement was seen during our experiments. The mechanisms behind these possible associations need to be further characterized. However, it may be postulated that evolutionarily-conserved mechanisms of expressional or metabolic “switching” may explain the differential susceptibilities among biofilm-positive and negative strains, aiming to accommodate bacteria to the dynamic changes observed in vivo. As a future aim, our studies could be extended to other relevant bacterial strains of importance in UTIs (e.g., *K. pneumoniae*, *S. aureus*, *S. saprophyticus*, *Enterococcus* spp.) where antibiotic resistance has shown to be a major concern, to ascertain a possible correlation of biofilm-formation and the susceptible/resistant phenotype. In addition, other budget-friendly experimental model systems may also be utilized in these studies.

In summary, our results have shown no relevant association between biofilm-production and resistance to several antibiotics, while ESBL-production was a predictor of lower prevalence of biofilm-producing strains. On the other hand, a significant, but not complete agreement was noted between biofilm-positivity and the phenotype of mucoid variant colonies.
